# An explainable end-to-end computer vision pipeline for detection, segmentation, and reconstruction of occluded weapons in forensic imagery

**DOI:** 10.3389/frai.2026.1867175

**Published:** 2026-07-23

**Authors:** Vaibhav Rohella, Kumar Anurag, Aditya Kumar, Manjula V., Shanthi P.

**Affiliations:** The School of Computer Science and Engineering, Vellore Institute of Technology, Chennai, India

**Keywords:** forensic AI, concealed weapon detection, RT-DETR, mobileSAM, stable diffusion, generative AI, crime scene analysis

## Abstract

**Introduction:**

Images from crime scenes often show partially concealed weapons due to obstructions such as hands and clothing, as well as surveillance camera limitations, which affect the efficacy of traditional detection methods. This work proposes an explainable forensic pipeline for occluded weapons detection, segmentation, and reconstruction.

**Methods:**

The proposed framework integrates RT-DETR-L, a transformer-based weapon detection model; MobileSAM for zero-shot segmentation of visible weapon regions; Stable Diffusion Inpainting for reconstruction of occluded regions; and a bilateral filter with Canny edge detection for forensic sketch generation. The detector was trained using synthetic occlusion augmentation on five weapon categories, such as firearm, grenade, knife, pistol, and rocket, and fire as an additional class for the environmental hazard indicator.

**Results:**

The RT-DETR-L model achieves a mAP@50 of 0.86 and a mAP@50-95 of 0.65, averaged over the five weapon classes, on a dataset with synthetic occlusion augmentation. Reconstruction quality meets minimum quality thresholds (SSIM > 0.54, PSNR > 16 dB, Region IoU > 0.80) up to approximately 50% occlusion. An interactive Streamlite application demonstrates the pipeline’s feasibility in 42–46 seconds as a controlled laboratory prototype.

**Discussion:**

The proposed explainable forensic pipeline highlights the potential of combining transformer-based detection with generative AI to assist forensic weapon analysis under challenging occlusion scenarios. The generated reconstructions and sketches are intended as visual aids for expert review and must undergo independent forensic validation before any operational use.

## 1 Introduction

Officers and investigators are faced with large quantities of video recorded by the cameras set up in public areas, transport means, educational institutions, as well as enterprises and organizations. The problem is that processing such information manually would be unfeasible; thus, there is a need for automatic weapon detection solutions that could contribute to security purposes as well as investigation. However, it is worth mentioning that in actual life weapons are never detected completely, as they usually hide behind hands, clothes, vehicles, furniture, or other objects. As a result, important information concerning trigger guard, barrel shape, slide mechanism, knife structure, grenade pins, among others, may remain unnoticed. Therefore, even advanced algorithms, such as YOLOv8 and Faster R-CNN, often struggle to detect these objects accurately (Thakur et al., 2024).

This problem has serious consequences from the point of view of the forensics study. In analyzing a video clip that shows a partially obscured gun, a forensics expert is supposed to recognize not only the presence of such a gun but also to know what kind of a gun this is and its general outline. Such knowledge is needed to estimate the danger and make a proper record of it. The problem with analyzing a video manually is that it takes much time and largely depends on the individual forensics officer’s expertise. It is made much more complicated by the numerous types of occlusion that can occur in practice. For instance, there is a possibility that an object will be partially occluded by being close to a body part, whereby the only visible component of the weapon will be its handle. In another case, a gun placed behind a car door may leave just the muzzle visible. There can also be instances where a weapon will be mostly occluded from view and visible only through a small part of it. This creates different kinds of partial visibility. It also means that a rigid method of detecting the object becomes problematic.

Another problem concerns the issue of the quality of the information itself. The images captured on surveillance cameras are subject to such factors as low resolution, blurring due to movement, dim lighting, and distortion caused by image compression. As it happens in practice, in some situations, the captured frames might contain no clear images of the object at all. Recently, there have been some breakthroughs in other fields of study that make it possible to address this task from an end-to-end perspective. Self-attention transformers such as RT-DETR (Zhao et al., 2023) do not work with patches of images but can attend to the entire image at once. Therefore, it becomes less of a challenge to tackle issues such as occlusions, where it would be useful to take into account data from a broader context. Furthermore, foundation segmentation models, including (Kirillov et al., 2023) and its mobile version, MobileSAM (Zhang et al., 2023). Enable the generation of pixel-wise masks from just simple bounding box prompts. As a result, it will become unnecessary to create large annotated datasets specifically for weapon recognition. Finally, recent progress in latent diffusion models (Rombach et al., 2022) greatly enhances inpainting capabilities of neural networks, particularly through the use of textual guidance.

An integrate all three technologies, together with a classical computer-vision sketch module, into a single forensic aid pipeline intended as a controlled research prototype. The first provide a brief overview of our system architecture and then describe how we went about creating a complete working system from start to finish. The creation of our AI forensic system involved four distinct phases:

1. Phase One—Development of an AI Processing Module (AIPM) to process forensic imagery.
2. Phase Two—Development of an AI Sketch Module (AISketch) to create a sketch of the subject(s) depicted in the forensic imagery.
3. Phase Three – Development of a Forensic Evidence Presentation Module (FEPM) that generates a report detailing how the forensic evidence was processed to create a photocopy-shaped sketch.
4. Phase Four – Validate that the AI-generated sketch meets evidential criteria for submission to a court.

The proposed forensic system will generate AI-assisted sketches as supporting evidence for legal proceedings. Outputs must be verified by qualified forensic experts due to strict reliability and admissibility requirements. The key challenge is creating an explainable and traceable framework that produces quality forensic sketches while preserving relevant artifacts.

## 2 Related work

### 2.1 Object detection

In the previous ten years object detection has made a lot of changes to meet the demands of both higher precision and quicker speeds. For a period of time CNN-based models were the leading methodology in the field, such as the Faster R-CNN (Ren et al., 2015)model that produced a two-stage process to detect and classify objects that is commonly used as a benchmark across various studies. The single-stage models developed after this, like the YOLO series (Jocher et al., 2023), offered a reduction in precision for an increase in speed. For example, YOLOv8 has approximately 53.9 mean average precision (mAP) on the COCO dataset but can do it at a rate of 479 frames per second (FPS) using a T4 GPU. Unfortunately, due to the limited field of view of convolutional models, partially occluded objects can be hard to detect; this issue is further complicated by needing to combine areas of the image that are far away from one another in order to identify such objects. For instance, if there is a weapon concealed behind another object, the model may only detect the weapon if it is able to connect visual cues from different areas within the image.

The recent advances in state-of-the-art object detection systems that have employed transformer architectures for end-to-end detection without reliance upon anchors or non-maximum suppression. The output from the DETR (Carion et al., 2020) model utilized learned set predictions via a bipartite matching loss function instead of relying on handcrafted post-processing for its output. Recent advancements with deformable DETR (Zhu et al., 2021) used limiting attention to a smaller amount of significant sampling points for each query, thereby effectively reducing the complexity of performing complete self-attention during training and inference. Further performance enhancements have been achieved with the RT-DETR (Zhao et al., 2023) model, which achieved a combined speed improvement of approximately 114 FPS along with a detection accuracy of 53.0 mAP within the COCO benchmark dataset. The significant improvement in speed and accuracy of the RT-DETR model can be attributed to its hybrid encoder architecture, which utilizes an intra-scale self-attention mechanism combined with efficient cross-scale feature fusion to enable the RT-DETR model to maintain strong global context knowledge whilst maintaining similar performance to that of previous state-of-the-art detection systems at higher speeds.

### 2.2 Foundation segmentation models

However, instance segmentation traditionally relied on dense annotations at the mask level. Mask R-CNN (He et al., 2017) improved upon Faster R-CNN with a parallel segmentation head based on ROI-Align, yet it suffers from the tight coupling of performance with the availability and richness of domain-specific training annotations. Collecting annotations such as masks at the pixel level for multiple weapon variants under varying degrees of occlusions would be prohibitively challenging for a forensic scenario.

SAM (Kirillov et al., 2023), trained on a set of 1 billion masks sourced from diverse internet imagery, shows that zero-shot segmentation of any object can be done using just a few prompts (e.g., bounding boxes, points, text) via a large Vision Transformer-based model. Although accurate, SAM’s performance comes at the cost of increased computational overhead associated with its ViT-H encoder model. MobileSAM (Zhang et al., 2023) tackles this problem by employing knowledge distillation to train a small ViT-Tiny backbone with a representation similar to the larger SAM architecture. This reduces model size from approximately 2.5 GB to 40 MB and inference time from 452 ms to 8 ms per image, with only marginal accuracy loss on box-prompted tasks—a compelling trade-off for our pipeline where segmentation must be fast to remain a practical intermediate step.

### 2.3 Generative image inpainting

Image inpainting—filling in missing or damaged parts of an image—has long been a core problem in computer vision. Early approaches based on patch matching (Criminisi et al., 2004) work by copying textures from surrounding regions into the missing area. Although this technique is quite effective in filling backgrounds or repeating patterns, it does not perform well when it comes to generating missing information which needs structure, like recreating the unseen portion of a weapon. The gated convolution and attention mechanism incorporated into GAN inpainting techniques (Yu et al., 2019) have solved this problem by allowing coherent generation of moderately sized holes. Nevertheless, such architecture can suffer from problems like mode collapse and instability during training phases. Diffusion-based generative models (Ho et al., 2020) have significantly advanced the field of image editing. These approaches create images via an iterative denoising process that begins with random noise and allows for much greater stability than is possible with GANs, all while creating images that have more diversity. This approach has been taken to the next step with latent diffusion models (Rombach et al., 2022), which conduct their computations within the latent space of a pretrained variational autoencoder. Additionally, conditioning mechanisms—such as cross-attention with CLIP-based text embedding allow the model to generate content aligned with specific classes described in natural language, which is central to our approach for reconstructing weapon-specific regions.

### 2.4 Weapon-specific detection work

Most existing research on automated weapon detection focuses on cases where the weapon is fully visible (Thakur et al., 2024). Current benchmark datasets also tend to lack systematic occlusion-based augmentation, and their evaluation methods usually do not distinguish between fully visible and partially hidden objects. To the best of our knowledge, no prior work combines transformer-based detection, zero-shot segmentation using foundation models, and class-guided generative inpainting into a single pipeline aimed at producing forensic sketches from partially occluded weapons. Our approach addresses this gap by introducing both a new methodological framework and an evaluation strategy aligned with forensic quality requirements.

## 3 Proposed methodology

**Figure 1:**
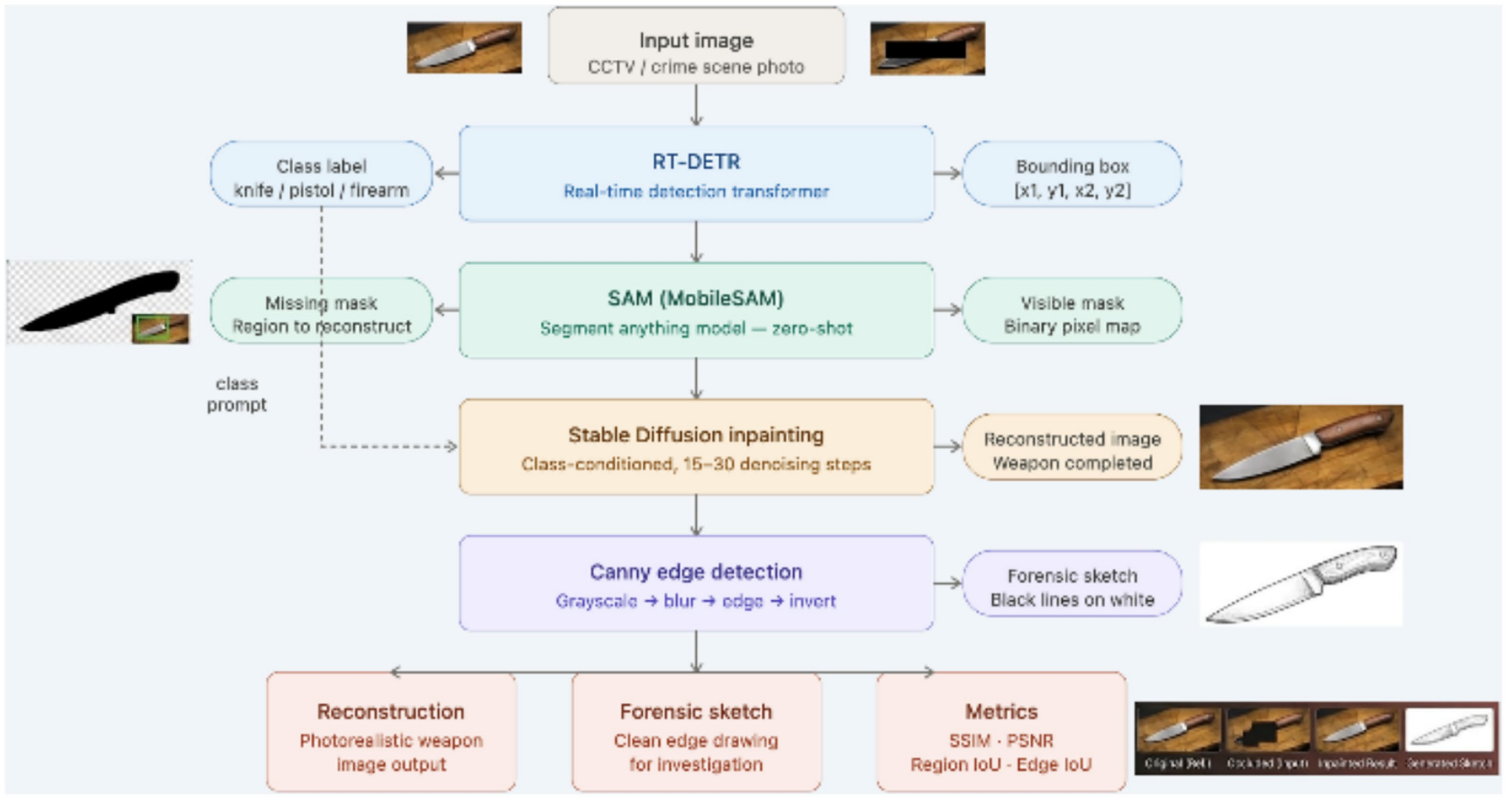
Five-stage forensic reconstruction pipeline.

Figure 1 illustrates the proposed system which consists five-step process that takes a single image input: first, RT-DETR detects the object; second, MobileSAM performs segmentation; third, an adaptive canvas generated; fourth, use Stable Diffusion to paint the object in; and finally, a forensic sketch generated. Every stage of this process can be computed as separate modules, allowing for a variety of alternative models to be swapped out easily within this same pipeline. Also, every step of computing will save the intermediate outputs generated, i.e., detection overlays, segmentation masks, and inpainting inputs and results.

These artifacts are useful for forensic review and also help with debugging and validation in real-world deployments. The modular design also permits flexible deployment: the detection and segmentation stages run efficiently on a consumer-grade GPU or even a powerful CPU, while the inpainting stage requires GPU memory of at least 8 GB. In resource-constrained field deployments, the sketch stage alone can be run on a previously generated reconstruction, decoupling the computationally expensive generation step from the time-sensitive sketch output.

### 3.1 Dataset preparation

**Figure 2:**
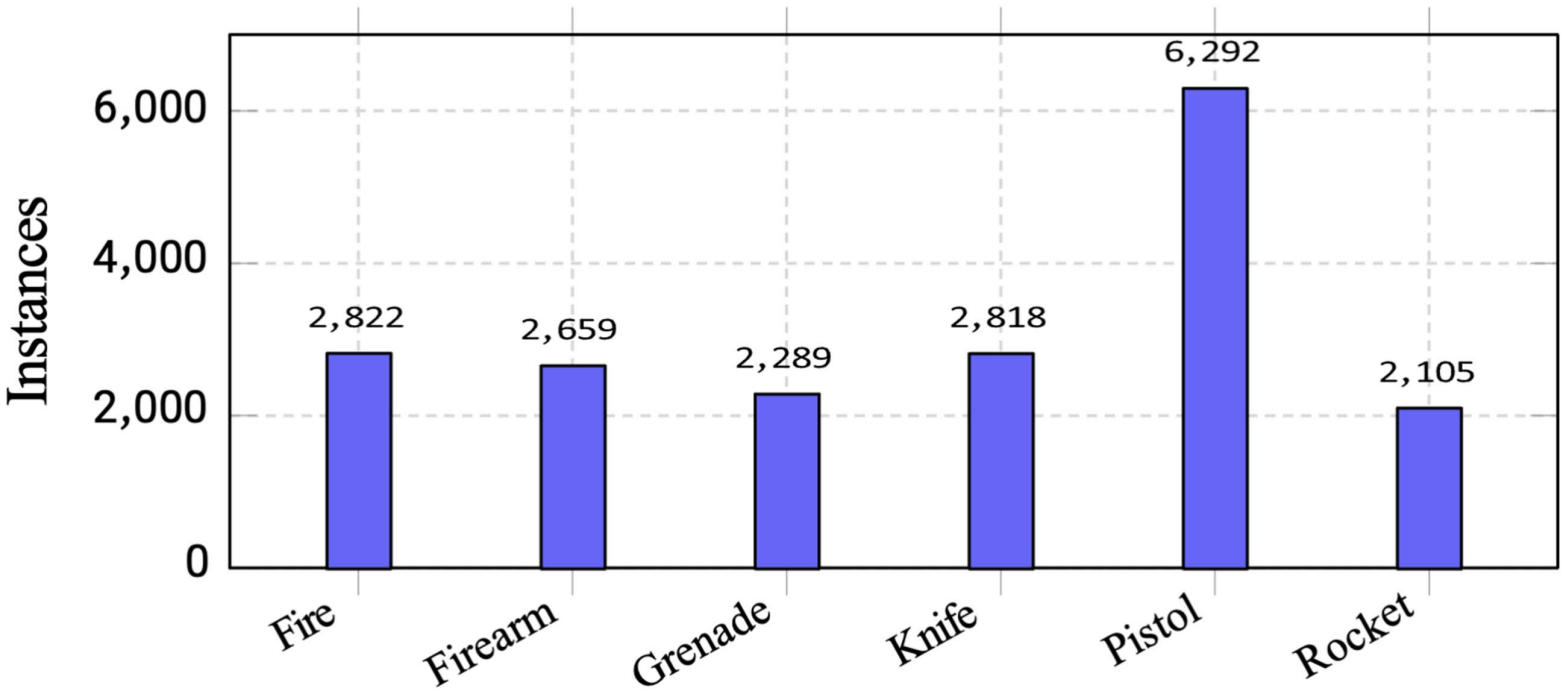
Dataset class distribution across six weapon categories.

The training corpus comprises six weapon categories: *fire*, *firearm*, *grenade*, *knife*, *pistol*, and *rocket*. Images were sourced from multiple publicly available repositories (Singh, 2023; Roboflow Universe, 2023; Roboflow Universe, 2023) and subjected to a standardised re-annotation process in YOLO label format to ensure consistent bounding-box conventions across sources. Duplicate images were removed using perceptual hashing, and images with resolution belo200 × 200 pixels were discarded to avoid introducing low-quality training samples. The resulting class distribution is shown in Figure 2, with pistol being the most represented class (6,292 instances) and rocket the least (2,105 instances).

An 80/10/10 train/validation/test split was applied using stratified sampling to preserve class proportions across all splits. To simulate real-world partial visibility, synthetic occlusion patches of varying size (10–80% of bounding-box area), shape (rectangular, elliptical, and irregular), and intensity (solid colour, Gaussian noise, and copied background texture) were procedurally overlaid on training and validation images. This occlusion augmentation scheme ensures that the detector encounters the full spectrum of practical partial-visibility scenarios during training, rather than only learning from clean, unclouded examples. Standard augmentations including random horizontal flipping, mosaic composition, and copy-paste were applied on top of the occlusion overlays. In compliance with SAGER guidelines, it is noted that the underlying dataset was sourced from publicly available repositories and includes surveillance images and videos in which humans may appear interacting with weapons. Sex and gender dimensions are not directly relevant to the technical objectives of this computer vision pipeline; the system operates on visual weapon morphology and does not consider or encode any human biological or social attributes in its detection, segmentation, or reconstruction stages.

### 3.2 Detection: RT-DETR-L

RT-DETR-L was selected as the detection backbone owing to its unique combination of real-time inference speed and transformer-level global context modelling. The architecture utilizes HGNetv2 convolutional network backbone to extract multi-scale features efficiently at three different output resolutions (P3, P4, P5) through stride 8, 16, and 32. These features are fed into a hybrid encoder that first applies intra-scale self-attention within each pyramid level to capture fine-grained spatial relationships, then performs cross-scale feature fusion via deformable attention to propagate context across scales. The decoder uses a query-selection strategy that initializes *K* = 300 object queries from the top-*K* high-confidence encoder positions, greatly accelerating convergence compared to randomly initialized queries in original DETR.

The model was initialised with weights pre-trained on COCO and fine-tuned on our six-class weapon dataset. Key training hyper parameters were Adam optimizer with an initial learning rate of 10^*−*3^, cosine annealing learning rate schedule over 100 epochs, early stopping with a patience of 15 epochs on validation mAP, and batch size of 16. The combined augmentation strategy described in Section 4.2 was applied online during training. No separate hyper-parameter search was conducted; the default RT-DETR-L configuration was used as a starting point, with the learning rate being the only hyper-parameter manually tuned via a brief grid search over {10^−2^, 10^−3^, 10^−4^}.

At inference time, a detection confidence threshold of 0.40 is applied, and the highest-confidence bounding box per image is forwarded to the segmentation stage. This single-detection policy reflects the forensic use case, where analysts typically focus on one weapon of interest at a time, though multi-detection support is discussed as a future extension.

### 3.3 Segmentation: MobileSAM

The bounding box produced by RT-DETR is padded uniformly by 20 pixels on each side before being passed to MobileSAM’s SamPredictor interface as a box prompt. This padding accommodates the slight under-prediction tendency of bounding-box detectors on partially occluded objects, ensuring that the segmentation mask can extend to the true weapon boundary even when the detector box is marginally tight. MobileSAM generates three candidate masks based on IoU confidence, and the one with the best score is selected.

This mask is then refined through a two-step post-processing stage. First, morphological closing with a 5 × 5 circular structuring element is applied to fill small gaps, which often occur due to light reflections on different weapon surfaces. Following that, any areas smaller than 200 pixels that may have been generated during this segmentation process are cropped out because they can most probably be attributed to background noise and not the actual weapon. This generates the binary mask image of the weapon region that is segmented. The inverse of the same is used as the inpainting mask in the next step.

### 3.4 Inpainting: stable diffusion

The inpainting stage employs the stable-diffusion-2-inpainting model (Rombach et al., 2022) accessed through the Hugging Face diffusers library. The model’s 9-channel UNet input comprises three components concatenated along the channel dimension: the 4-channel noisy latent representation of the target image, the 4-channel VAE-encoded visible image in which the occluded region has been zeroed out, and the 1-channel binary mask down sampled to the latent resolution. This concatenation scheme allows the network to simultaneously attend to the known image context and the mask geometry when denoising the masked latent.

An adaptive canvas cropping step is applied before inpainting. Rather than processing the full input image at the model’s native 512 × 512 resolution, which would reduce weapon detail in high-resolution images, the padded bounding-box region is cropped, resized to 512×512, inpainted, and then composited back into the original image. This setup helps maintain the highest possible resolution for the weapon and its surrounding region within the model’s fixed input size. Class-specific positive prompts (Table A1 in Appendix) will be used to guide the generation process, reinforce the weapon type, ensure it is completely reconstructed, and maintain a clean image with respect to forensic use. A negative prompt (blurry, distorted, low-quality, duplicative, with a watermark, with extra limbs, deformed) will be used for every case to limit common diffusion model artifacts. The DDIM algorithm will be used for sampling with 30 inference steps and classifier-free guidance of 7.5, which balances output quality and throughput efficiency. An inpainting operation typically takes 7–10 seconds using an NVIDIA RTX 3050 (6GB VRAM). Inference time can further be reduced through the use of half-precision (FP16) inference, which does not affect the quality of the results.

### 3.5 Forensic sketch generation

The final step in the process is the creation of a forensic line drawing from the photorealistic reconstruction that can be inserted into police investigative documents. The first step is to convert the recovered weapon image from RGB color to grayscale. The second step is to apply a bilateral filter using the following parameters: *d* = 9, *σ*_color_ = 75, and *σ*_space_ = 75. This will produce a blurred/ringed effect in the image inside the weapon outline but will not produce a blurred/ringed effect outside the weapon outline, which is exactly what is needed for the weapon outline. The next step involves Canny edge detection with thresholds of 50 and 150, respectively. The binary map of edges is subsequently subjected to dilation using a 3 × 3 kernel and inversion to create black line drawings against the white background characteristic of technical illustrations. This stage requires no trained model and executes in under one second, making it the most computationally efficient stage of the pipeline. The choice of classical computer vision rather than a learned sketch synthesis model is deliberate: it eliminates any additional generative model from the chain, reducing the number of sources of potential hallucination or distortion in the final output.

## 4 Experiments and results

### 4.1 Experimental setup

All experiments were conducted on a workstation running Windows 11 with an NVIDIA RTX 3050 GPU (6 GB VRAM), an AMD Ryzen 77840HS CPU, and 16 GB of DDR5 system RAM. RT-DETR-L was trained using the Ultralytics frame-work. MobileSAM inference used the official MobileSAM repository. Stable Diffusion inpainting used Hugging Face diffusers v0.24 with FP32 precision. Evaluation metrics were computed using standard implementations from scikit-image (SSIM, PSNR) and custom IoU routines. All reported numbers are averaged over the held-out test split unless stated otherwise.

### 4.2 Detection performance

Table 1 summarises per-class and mean detection performance on the test split. Pistol achieves the highest per-class mAP@50 of 0.91, which can be attributed to its compact, rotationally symmetric silhouette and the large number of training instances available for this class. Knife and firearm also perform well (mAP@50 of 0.89 and 0.87 respectively), owing to their distinctive elongated profiles. Grenade and rocket score lower, reflecting the challenges posed by high shape variability across grenade subtypes and limited training samples for rockets, respectively.

**Table 1:** Per-class detection performance on test split.

Class	mAP@50	mAP@50–95	Remarks
Firearm	0.87	0.66	Distinct barrel shape
Grenade	0.82	0.61	High shape variation
Knife	0.89	0.68	Compact profile
Pistol	0.91	0.70	Best per-class AP
Rocket	0.81	0.60	Few training samples
Mean	0.86	0.65	Excl. fire

The dataset comprises six categories in total: five weapon classes (firearm, grenade, knife, pistol, rocket) and fire, which is treated as a separate environmental hazard indicator rather than a weapon category. Fire detection is not included in the mean accuracy computations reported in Table 1, as this class is not part of the weapon-reconstruction pipeline. However, the model demonstrates strong fire detection performance when present.

**Figure 3:**
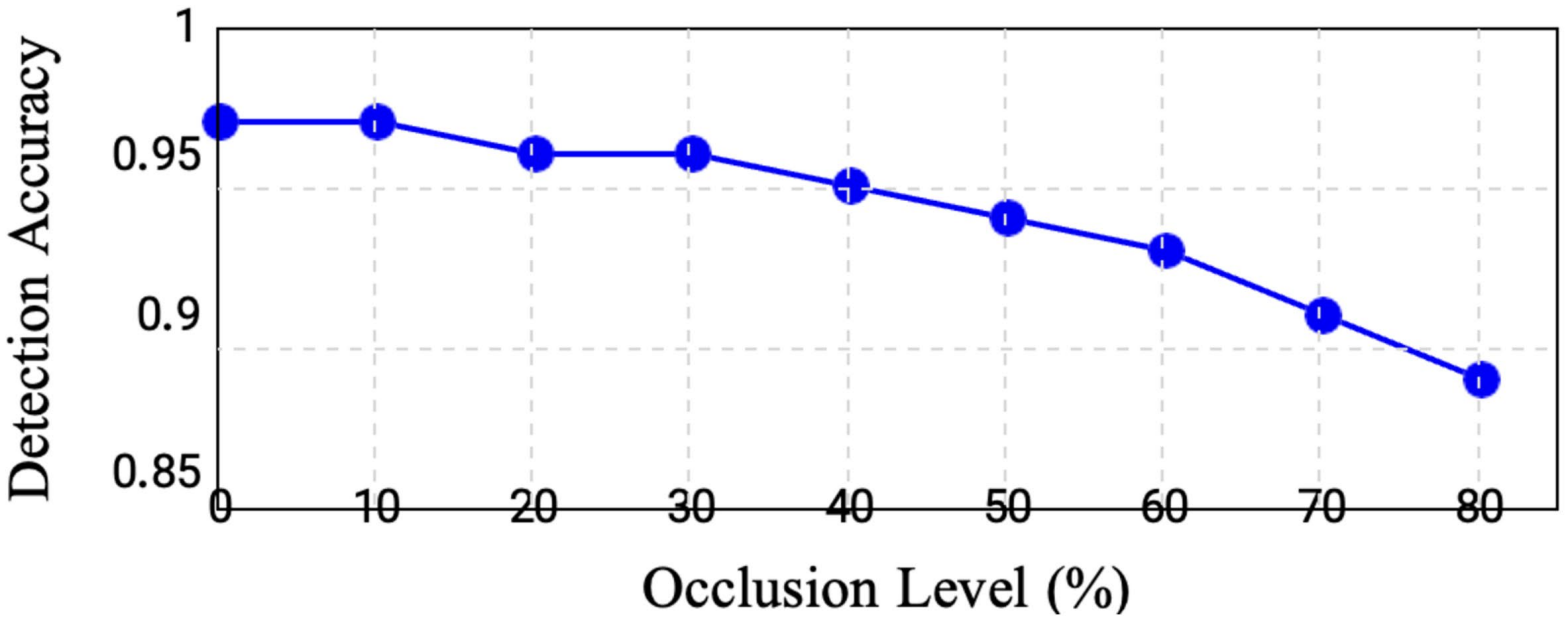
Detection accuracy remains robust across occlusion levels 0–80%.

Figure 3 illustrates the variations in detection accuracy when the level of occlusion increases from 0 to 80%, evaluated over the full held-out test split (approximately 1,700 images stratified across six classes). Error bars in the figure indicate one standard deviation calculated across five random subsamples of the test split. Notably, the accuracy remains above 0.88 even at high occlusion levels, leveraging the transformer model’s ability to use global context—such as hand positioning, scene background, and partial visual cues. In contrast, traditional CNN-based detectors often struggle to exploit effectively such long-range contextual dependencies under highly occluded scenarios.

### 4.3 Segmentation quality

**Figure 4:**
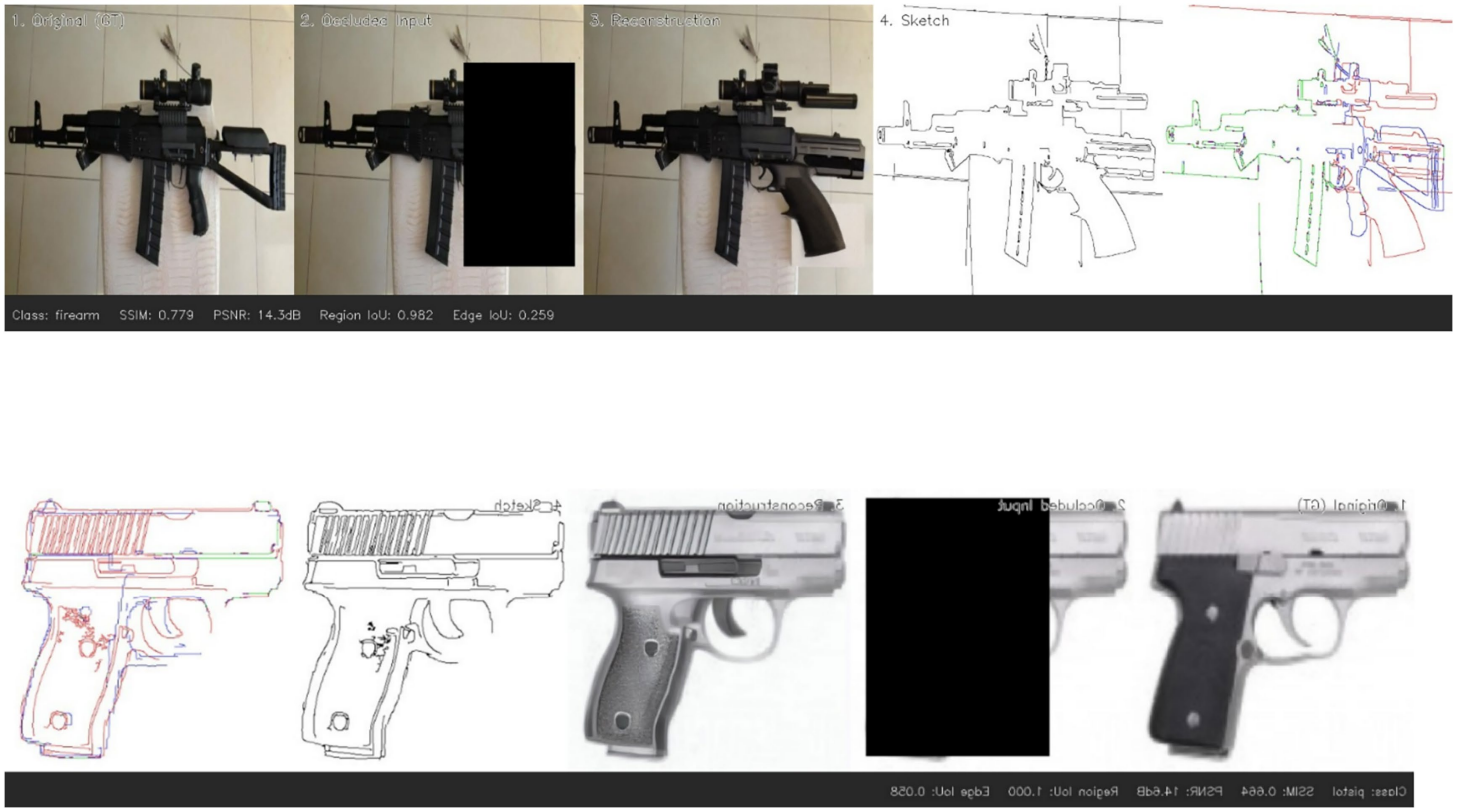
Image inpainting and sketch reconstruction results for firearm images.

Table 2 shows per-class MobileSAM segmentation performance. On clear, unoccluded images, the overall mean IoU is 0.81 and mean Dice coefficient is 0.89. On occluded images, these values drop to approximately 0.75 and 0.84 respectively—a modest degradation that is within acceptable bounds for a zero-shot method that has never seen weapon-specific segmentation annotations. The SAM (Shanthi and Manjula, 2025) confidence scores (predicted IoU) also remain high across all classes, suggesting that the model’s internal confidence estimation is reasonably calibrated for this domain despite the domain shift from natural image training data and shown in Figure 4.

**Table 2:** Per-class MobileSAM segmentation performance.

Class	Mean IoU	Mean Dice	SAM score
Pistol	0.84	0.91	0.92
Knife	0.85	0.92	0.93
Firearm	0.79	0.88	0.88
Grenade	0.77	0.87	0.87
Rocket	0.81	0.89	0.90
Overall	0.81	0.89	0.90

The ‘knife’ class achieves the highest IoU score (0.85), likely because its elongated shape and strong contrast against the background make it easier to segment accurately. In contrast, grenades show the lowest IoU (0.77), which aligns with the detection results. This is probably due to the wide variation in grenade shapes, including cylindrical, pineapple-style, and stick designs, making consistent localization more challenging.

### 4.4 Reconstruction quality

**Figure 5:**
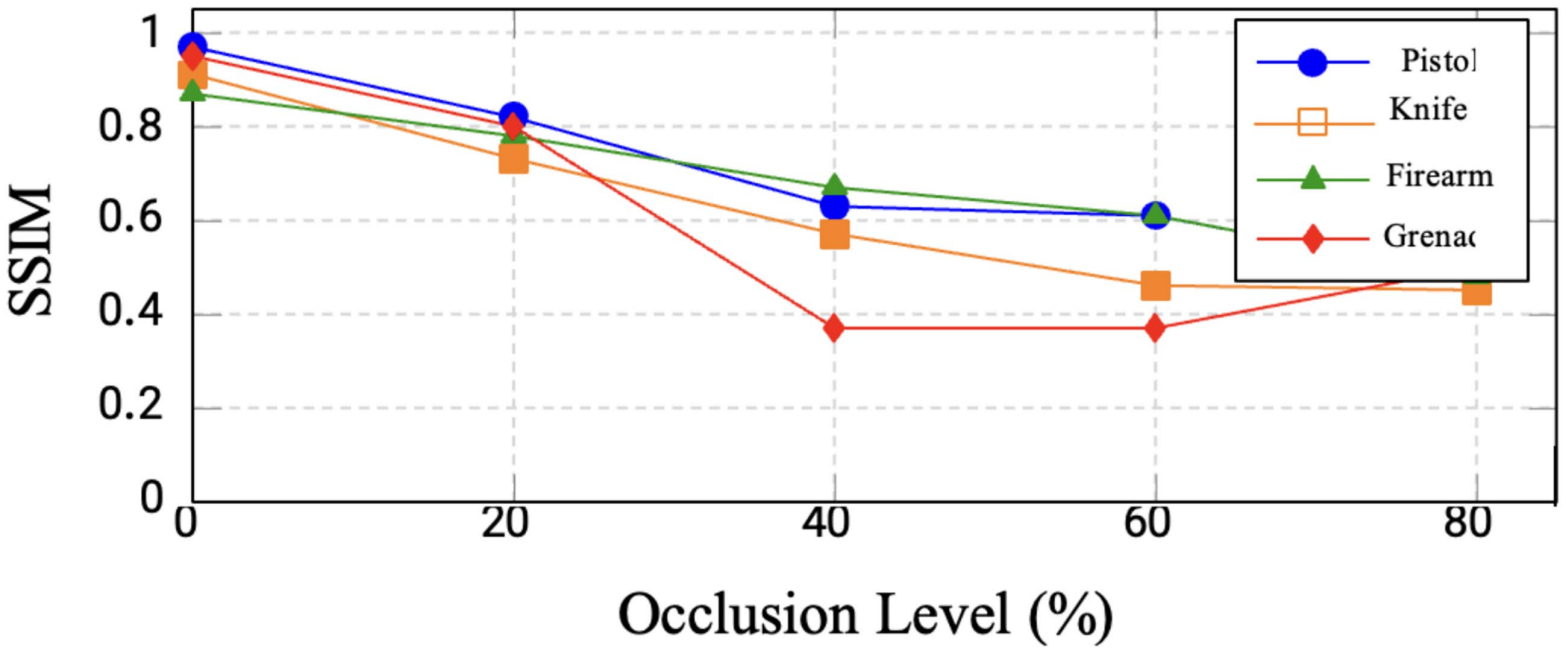
SSIM vs. occlusion level per weapon class. Generative inpainting maintains structural fidelity up to approximately 50% occlusion. Shaded bands denote ±1 standard deviation computed across five random subsamples of the test split.

**Figure 6:**
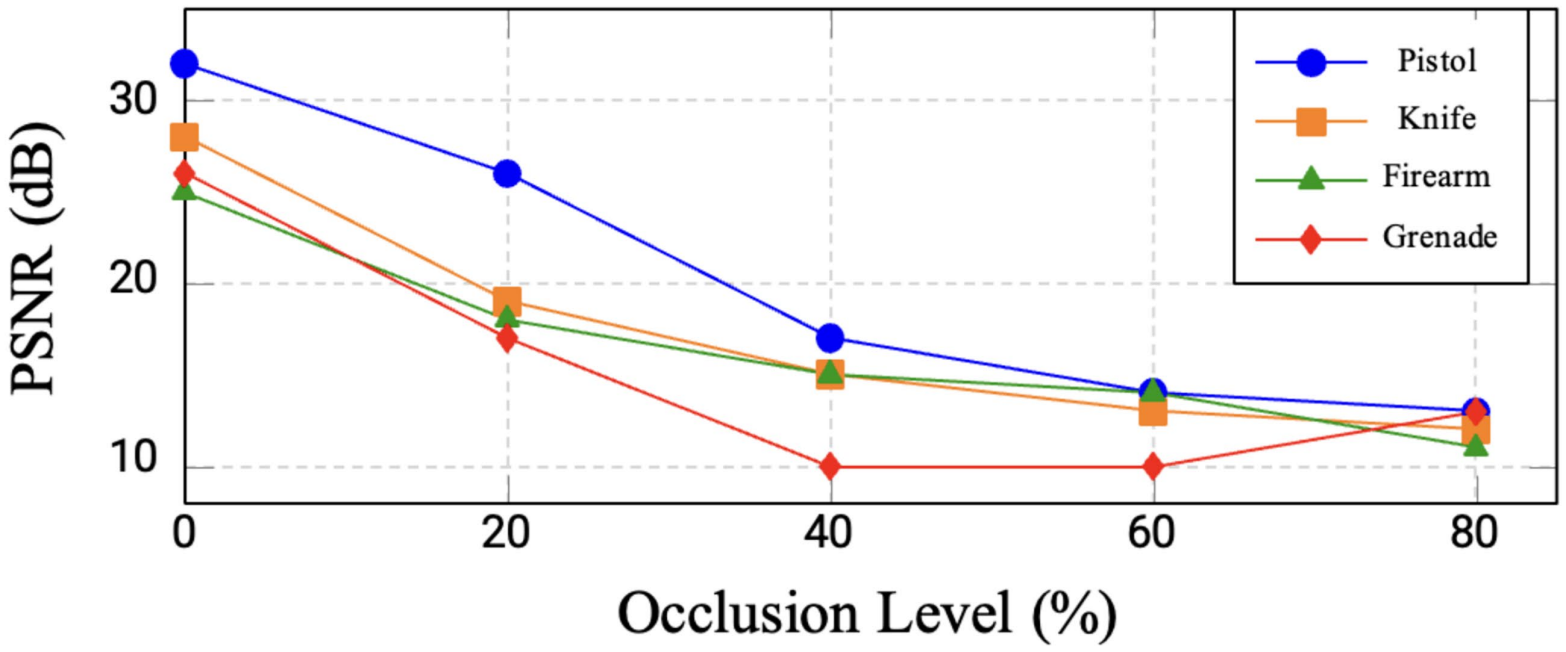
PSNR vs. occlusion level. Decline reflects the generative nature of inpainting rather than reconstruction failure. Shaded bands denote ±1 standard deviation computed across five random subsamples of the test split.

Figures 5, 6 present SSIM and PSNR values across different levels of occlusion for four weapon categories. As expected, both metrics gradually decrease as occlusion increases. When a larger portion of the image needs to be reconstructed, the model has to infer more missing information, making it harder to closely match the original image at the pixel level.

A forensic threshold of SSIM > 0.54 and PSNR > 16 dB is achieved up to approximately 50% occlusion across most weapon classes, which we consider a practically relevant operating regime. At occlusion levels beyond 50%, metric values decline more steeply; however, region-level IoU (Section 5.5) remains above 0.80, confirming that the re-constructed weapon’s spatial extent and general morphology remain correct even when fine texture detail diverges from the ground truth.

**Figure 7:**
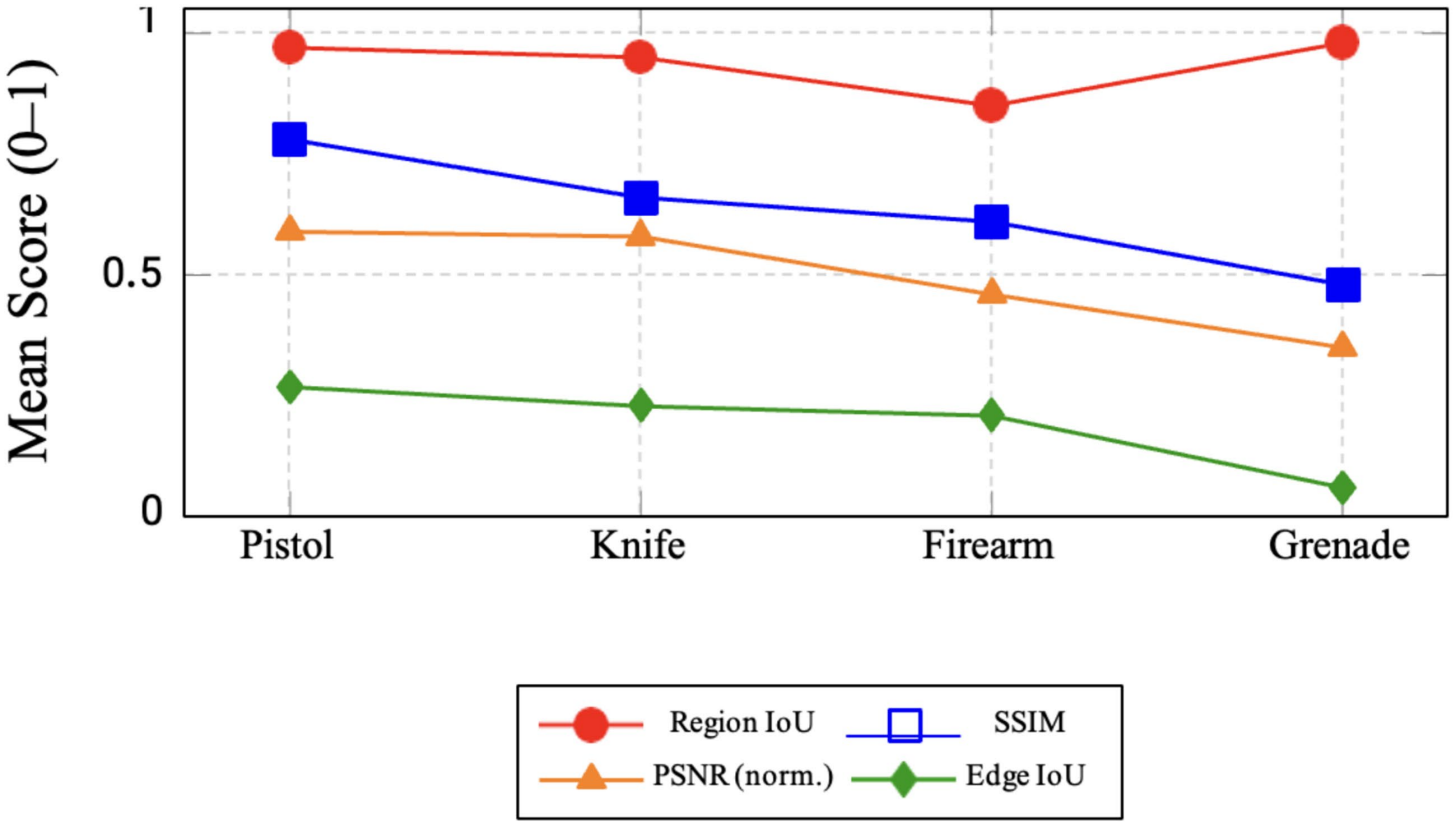
Mean metric scores per weapon class. Region IoU remains consistently high while Edge IoU reveals greater variability for complex shapes.

As shown in Figure 7, a combined metric summary is reported for each weapon class, averaged across all occlusion levels. Given that the Region IoU scores were relatively high, ranging from 0.85 to 0.98, it is evident that the model can recover the general form and shape of the silhouette of the weapons. On the other hand, the Edge IoU scores are low, especially those of grenades, owing to difficulties in recreating surface features. These finer details are less critical from a forensic standpoint, though improving class-specific inpainting could help address this limitation.

### 4.5 End-to-end pipeline evaluation

The complete pipeline was evaluated on 20 held-out images (4 images per class at three occlusion levels: 25, 50, and 75%) that were not used in any training or validation step. Detection accuracy was around 90% at 25% occlusion and decreased steadily to approximately 68% at 75%, following the same downward trend observed in the per-class evaluation. The lower performance observed in this evaluation compared with the main detection performance analysis is attributed to the more challenging and cross-category composition of this image set (see Section 5.2 for a full explanation of the difference). Among the cases where detection succeeded, segmentation produced a clean and morphologically valid mask in 88% of instances. Overall plausibility—defined as correctly identifying the weapon type, maintaining realistic proportions, and preserving consistent geometry—was estimated at approximately 85% based on an internal pilot assessment by the authors. This plausibility score has not been validated by independent forensic experts and should be treated as a preliminary internal indicator rather than a confirmed forensic quality measure. It is reported here as a structured labeling exercise, not as validated ground truth.

**Figure 8:**
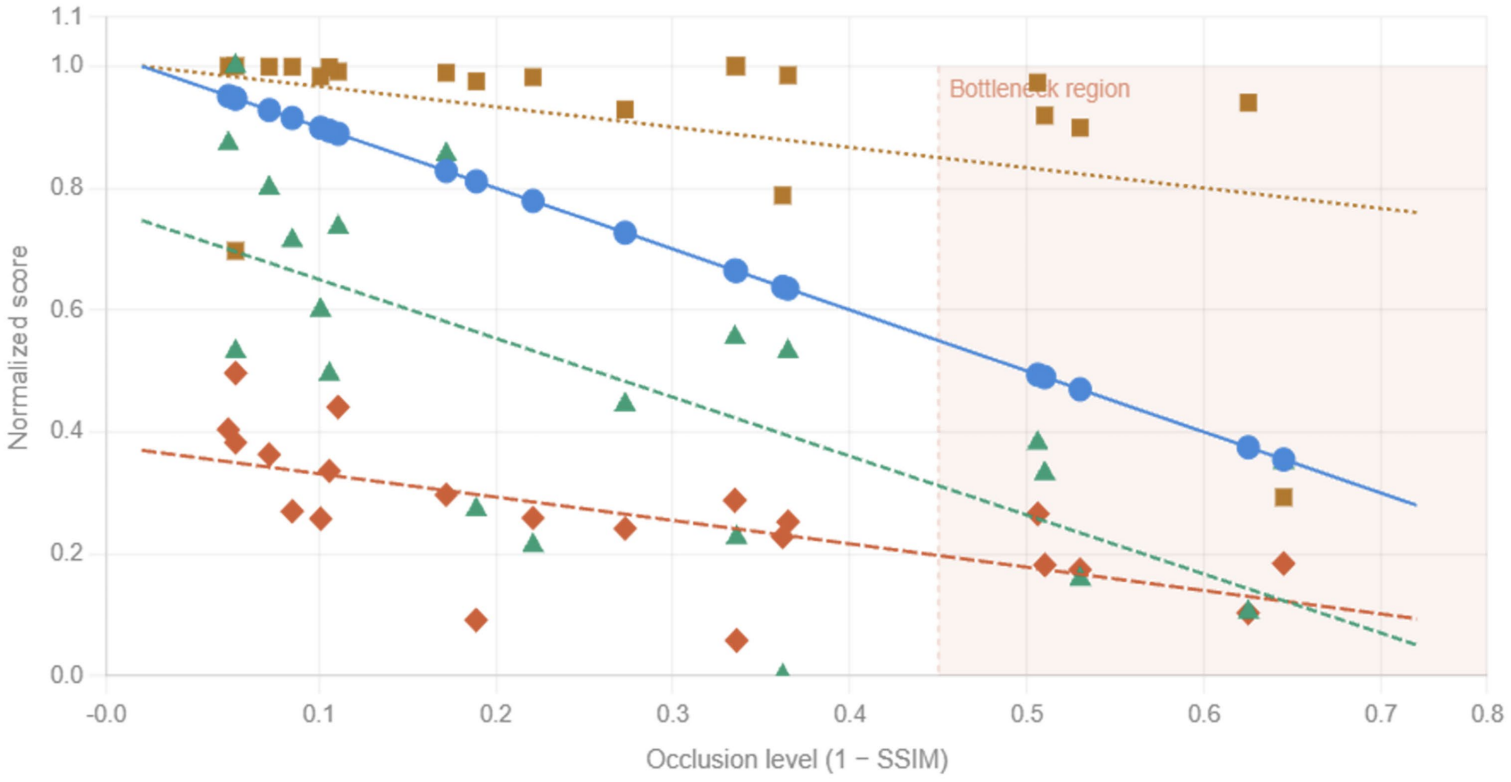
End-to-end reconstruction accuracy vs. occlusion level; the reconstruction stage is the primary pipeline bottleneck at high occlusion levels.

Reconstruction takes up most of the execution time, taking 38 to 42 s per image with a RTX 3060 in FP32 precision as shown in Figure 8. Image detection and segmentation require only about three seconds in total. Image synthesis takes less than one second. As such, the entire process takes roughly 42 to 46 s per image, not instantly but still good enough for a forensics lab, where analyst time rather than computation time becomes the limiting factor.

### 4.6 Comparison with baseline methods

The ablation study was conducted by comparing the proposed system with its four progressively simplified variants presented in the Table 3. Table III compares the proposed system against four progressively simplified variants. The baseline in row 2 (YOLO + Box Prompt + Generic Inpainting) represents a minimal integration where none of the three key design choices—transformer detection, foundation segmentation, and class-constrained generation—are present. Each subsequent row adds one of these elements. Removing class-specific prompts (row 3) causes the largest single drop in class accuracy, confirming that guided generation is the most critical individual design decision. Replacing MobileSAM with a simple bounding-box crop as the inpainting mask (row 4) results in a moderate reduction in segmentation quality and introduces background pixels into the reconstruction region. The LaMa baseline (row 5) demonstrates that without class-conditioned generation, even high-quality inpainting architecture cannot produce weapon-class-consistent completions.

**Table 3:** Comparison of Pipeline Variants and Baseline Methods.

Method	Detection	Segmentation	Class Accuracy	Remarks
Proposed pipeline	High	High	High	Full system
YOLO + Box + Generic	Medium	Low	Low	Basic setup
YOLO + SAM + Generic	Medium	High	Medium	No class guidance
RT-DETR + Box + Class prompt	High	Low	Medium	No segmentation
LaMa inpainting	High	High	Low	No class awareness

## 5 Discussion

### 5.1 Strengths and design justification

The transformer-based RT-DETR detector handles partial occlusion more effectively than comparable CNN-based models because it uses a global self-attention mechanism, allowing it to consider context from the entire image rather than relying only on local features. Unlike convolutional networks that can only aggregate local features within a limited receptive field, the transformer encoder attends to all spatial positions simultaneously, enabling detection cues to propagate across the full image extent. This is particularly important for heavily occluded weapons, where the only visible evidence may be a small, isolated fragment that is interpretable only in the context of the surrounding scene. Zero-shot MobileSAM segmentation eliminates what would otherwise be a significant annotation bottleneck. Collecting dense pixel-level masks for weapons across multiple classes, occlusion levels, lighting conditions, and viewing angles would require substantial expert annotator time and introduce annotation inconsistencies. The ability to rely on box-prompted foundation segmentation without any domain-specific training data is therefore a practically important property of the proposed system.

Class-conditioned Stable Diffusion inpainting is one of the key contributions of the pipeline and arguably its most impactful component. In either of the traditional frameworks whether patch-based or GAN-based, there is no guarantee for achieving any form of consistency in terms of ensuring that the reconstructed scene will match the weapon category detected. This problem can be solved by conditioning the generated prompts using the detected weapon category from the detector. In doing so, the scene reconstruction becomes semantically consistent in addition to being visually realistic.

### 5.2 Limitations

There are certain drawbacks in the present model that must be admitted. For instance, the failure in detecting occlusions greater than 75 percent occurs continuously within the pipeline, as all the successive phases rely on the correct formation of the bounding box. The next task is to devise a backup plan that could be used in case of excessive occlusions by taking into account the categorization of scenes instead of directly detecting weapons. Secondly, it takes around 40 s to reconstruct a single picture, which rules out the possibility of a real-time application. Thirdly, conventional metrics based on pixel similarity to a reference image, such as SSIM and PSNR, do not accurately assess the quality of perception of the generative outputs since an equally valid output of a generator, though different from the target, would result in poor scores for this metric. In order to more accurately evaluate quality in this case, either Fréchet Inception Distance (FID) or expert evaluation would be more applicable to the process of inpainting. Fourthly, the algorithm currently processes only one high-confidence detection for each frame, thus overlooking potentially other weapons in crowded settings.

### 5.3 Ethical considerations

Forensic use of AI generated imagery comes with its own set of ethical considerations. Several security measures have been introduced in the present design to avoid misuse. Processing is entirely carried out at the local machine of the user/analyst and there is absolutely no uploading or cloud API integration, which protects the confidentiality of evidence against possible data breach. All reconstructions and sketches generated are automatically embedded with an “AI Generated Forensic Aid” watermark to differentiate them from original evidence or manually produced sketches. It is important to note that this project aims to provide auxiliary support for decision making and cannot be considered as autonomous forensic evidence. Generated reconstructions and sketches must not be used as primary evidence of weapon type, hidden structure, or culpability without independent corroboration and expert forensic interpretation. Any such output presented in legal or investigative proceedings must be clearly disclosed as AI-generated and reviewed by a qualified forensic professional.

## 6 Conclusion

In this paper, we have demonstrated a full pipeline that combines RT-DETR-L for weapon detection using transformers, MobileSAM for zero-shot pixel-wise segmentation, Stable Diffusion for class-aware inpainting, and classical bilateral filtering + Canny edge detector for the generation of line sketches. With this combination of four stages, our pipeline can detect, segment, reconstruct, and generate forensic documents for partially occluded weapons in a single image input, thereby fulfilling a functionality gap that has not been addressed by traditional computer vision systems. Results have shown that our system scores mAP@50 = 0.86 for detection of five weapon classes, mean IoU = 0.81 for zero-shot segmentation, SSIM > 0.54 and Region IoU > 0.80 for reconstructions up to approximately 50% occlusion, and produces forensic line sketches without requiring a trained generative sketch model. Baseline comparisons demonstrate the effectiveness of each pipeline stage, with class-constrained generation being the most influential design decision. All outputs are intended as visual aids to support expert review, not as validated forensic evidence, and the system requires independent forensic validation before any operational use.

The inherent problems of the current architecture—namely, high reconstruction delay, single detection strategy, and the absence of formalized expert user validation—point the way forward in terms of future directions. We will explore flow-based inpainting systems for fast inference, integrate support for multi-object reconstruction with detection queue prioritization, introduce video temporal consistency using cross-frame attention or flow-guided inpainting, and finally, conduct a formal expert validation experiment involving working forensic analysts. Fine-tuning the reconstruction algorithm on a specialized weapons reconstruction dataset might significantly boost Edge IoU scores and reduce hallucinations when reconstructing structurally intricate weapons like hand grenades. We consider our approach a valuable contribution toward an explainable and usable AI-driven forensic aid system. This work presents a controlled research prototype; independent forensic validation by domain experts is required before the system can be considered for any operational deployment. The codebase and Streamlit application will be made available upon acceptance at a permanent repository (link to be provided in the final version), to support future research and reproducibility.

## Data Availability

The original contributions presented in the study are included in the article/supplementary material, further inquiries can be directed to the corresponding author.

## Appendix

**Table A1:** Positive prompts used per weapon class.

Class prompt (truncated)
Pistol “*complete realistic pistol handgun, full weapon visible, clean neutral background, forensic technical illustration...*”
Firearm “*complete realistic firearm rifle assault weapon, full weapon visible, forensic technical drawing...*”
Knife “*complete realistic knife blade, full weapon visible, clean white background, forensic technical drawing...*”
Grenade “*complete realistic hand grenade, full object visible, clean white background, forensic drawing...*”
Rocket “*complete realistic rocket launcher missile, full weapon visible, clean background...*”
